# *Pogostemon cablin* essential oil affects anxiety- and depressive-like behaviors and the gut microbiota in chronic unpredictable mild stress model rats

**DOI:** 10.3389/fnut.2024.1303002

**Published:** 2024-02-14

**Authors:** Puyue Ouyang, Dali Kang, Weijing You, Xiaozhong Shen, Xiaolu Mo, Yao Liu

**Affiliations:** ^1^College of Chinese Materia Medica, Guangdong Food and Drug Vocational College, Guangzhou, China; ^2^School of Traditional Chinese Medicine, Shenyang Medical College, Shenyang, China; ^3^College of Medical Technology, Ningbo College of Health Sciences, Ningbo, China

**Keywords:** *Pogostemon cablin*, essential oil, microbiota, gut–brain axis, chronic unpredictable mild stress

## Abstract

The gut microbiota is thought to be an important factor that influences brain processes and behaviors through the gut–brain axis. *Pogostemon cablin* is used in traditional Chinese medicine (TCM) to treat gastrointestinal symptoms. Patchouli essential oil (PCO), the main active agent in *P. cablin*, is used in aromatherapy for stress relief. The aim of our study was to investigate the effects of orally administered PCO on anxiety- and depressive-like behaviors and the gut microbiota. We constructed a rat model of chronic unpredictable mild stress (CUMS) and explored the anxiolytic- and antidepressant-like effects of PCO using the open field test (OFT) and forced swim test (FST). Changes in the abundance of the gut microbiota, short-chain fatty acids (SCFAs), and other related molecules were assessed to determine the role of the gut microbiota. Our results showed that CUMS induced an anxiety-like phenotype in the OFT, which was reversed by PCO, and that PCO also significantly mitigated the depression-like behaviors caused by CUMS in the FST. Furthermore, we found that PCO increased the relative abundances of several probiotics, including *Bacteroides* and *Blautia*, and decreased the relative abundances of *Ruminococcus_1* and *Ruminococcus_2*, which were increased by CUMS. Regarding SCFAs, the metabolites of the gut microbiota, PCO increased the concentration of propionic acid and decreased that of caproic acid. Finally, PCO restored the serotonin (5-hydroxytryptamine, 5-HT) level in the hippocampus, which had been decreased by CUMS. The results of this study suggested that PCO can improve stress-related anxiety- and depression-like behaviors and might exert its effects on the central nervous system through interactions with the gut microbiota.

## Introduction

*Pogostemon cablin* (Blanco) Benth. is a frequently utilized herbal medicinal product in traditional Chinese medicine (TCM). It is commonly used for treating gastrointestinal infectious disorders, including gastrointestinal cold, acute gastroenteritis, nausea, vomiting, and diarrhea. These conditions are believed to be linked to dampness and summer heat, as per the principles of TCM (1). *P. cablin* belongs to the Labiatae family, and patchouli essential oil (PCO) is considered its main effective component. Essential oils derived from plants are popular because of their positive emotional effects on calming nerves. PCO is also commonly used in aromatherapy for stress relief (2, 3). Stress is a significant contributing factor to the development of anxiety and depression, both of which are highly prevalent in the current world. However, initial treatment for these two disorders often results in adverse effects, such as loss of appetite, weight loss, drowsiness, dizziness, fatigue, and sexual dysfunction (4, 5). Therefore, the development of new psychopharmacological agents that have minimal adverse effects is urgently needed.

The importance of natural essential oils has increased due to their rich bioactivities and low risk of toxicity. Modern studies have revealed that PCO possesses antibacterial, antifungal, antioxidant, analgesic, and anti-inflammatory activities (2). However, to the best of our knowledge, the potential effects of PCO on anxiety and depression are still unknown. Accumulating evidence suggests a connection between the gut microbiota and the brain axis in terms of stress-related mental disorders, such as anxiety and depression (6). Research has shown that prebiotics have anxiolytic-like effects (7). Additionally, antidepressant treatments can impact the composition of the gut microbiota (8). Furthermore, essential oils can modulate the gut microbiota, thereby enhancing health and performance (9–12).

In this study, we established a rat model of chronic unpredictable mild stress (CUMS) and investigated the anxiolytic and antidepressant effects of PCO using the open field test (OFT) and the forced swim test (FST). To gain a deeper understanding of how PCO affects the central nervous system through modulation of the gut microbiota, we conducted 16S rRNA gene sequencing analysis. This allowed us to compare the differences in the taxonomic composition of the gut microbial community. Furthermore, the microbiota in the intestine plays a crucial role in metabolizing carbohydrates, resulting in the production of short-chain fatty acids (SCFAs). SCFAs have been found to be associated with the secretion of serotonin (5-hydroxytryptamine, 5-HT), and altered 5-HT has been implicated in the development of anxiety and depression (13). Furthermore, the gut microbiota can influence behaviors by regulating important molecules, including brain-derived neurotrophic factor (BDNF) and corticosterone (14). Therefore, we also examined the production of SCFAs and the levels of 5-HT, BDNF, and corticosterone. The results of this study may contribute to improving clinical applications and further pharmacological studies of PCO.

## Materials and methods

### Materials

Fluoxetine hydrochloride (Pathcon, France) was used as a reference drug. 5-HT and BDNF ELISA kits and a corticosterone immunoassay kit were purchased from Nanjing Jiancheng Bioengineering Institute (Nanjing, China). PCR amplicons purified from Agencourt AMPure Beads and a Quantifying PicoGreen dsDNA Assay Kit were purchased from Beckman Coulter (Indianapolis, United States) and Thermo Fisher Scientific (Carlsbad, United States), respectively. The Sequencing MiSeq Reagent Kit (v3) was purchased from Shanghai Personal Biotechnology Co., Ltd. (Shanghai, China). Saline (0.9%) was purchased from Qingdao Hope Bio-Technology Company (Qingdao, China). All other reagents and chemicals used were of analytical grade.

### Preparation and GC-MS analysis of patchouli essential oil (PCO)

*P. cablin* was collected from the Medicinal Plant Garden of Guangdong Food and Drug Vocational College. The identity of the plant was confirmed with morphological evaluations and comparison to reference data. A voucher specimen of the plant (IBSC38pc15) was deposited in the Herbarium of Guangdong Food and Drug Vocational College for identification. The aerial part of *P. cablin* was dried in the shade and powdered. PCO was obtained by hydrodistillation for 3 h using a modified Clevenger system. The fraction of essential oil was separated by density, with a yield of 2.0% (g/g crude drug).

PCO was analyzed by GC-MS (Thermo Fisher) instrument using a TR column (30 m × 0.25 mm × 0.25 μm). Constant flow at 1 ml/min of carrier gas was used for sample analysis. The injector temperature of the instrument was programmed at 220°C. Oven temperature was started from 50°C to 250°C with ramp of 4°C/min. Sample was injected in a split mode (1:50) with injection volume of 1 μl. Temperature of the ion source was set at 220°C and transfer line temperature was at 300°C and ionization of the sample was performed in electron impact mode at an ionization voltage of 70 eV with mass range used from m/z 50 to 650 amu. The identification of the essential oil compounds was based on comparing mass spectra of compounds (NIST and WILEY libraty).

### Animals and treatment

**Figure 1:**
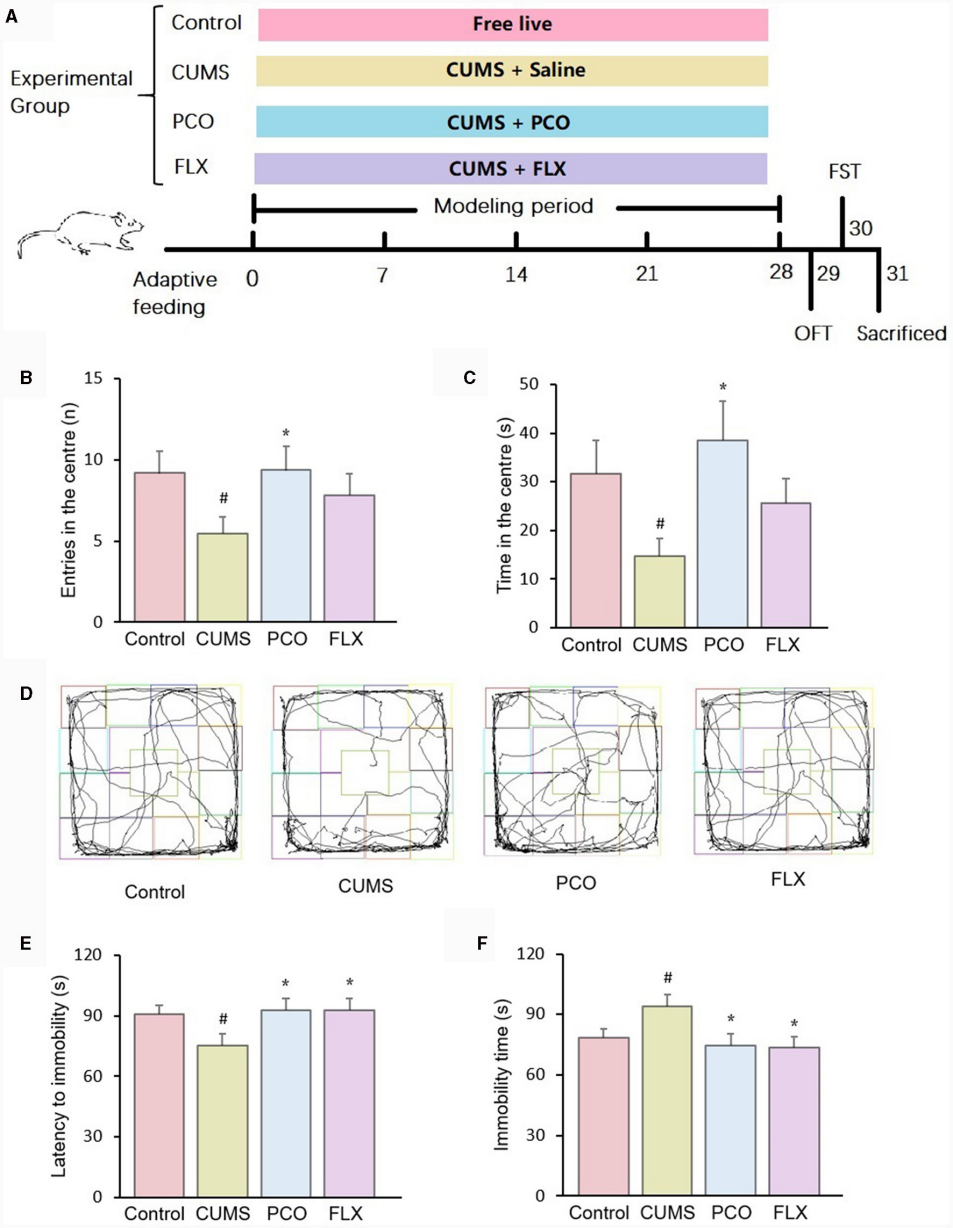
Effects of patchouli essential oil (PCO) on the behaviors of rats with chronic unpredictable mild stress (CUMS). **(A)** Schematic illustration of the animal experimental design. **(B)** Number of entries in the center in the open field test (OFT). **(C)** Time spent in the center in the OFT. **(D)** Representative track sheets of the OFT. **(E)** Latency immobility in the forced swim test (FST). **(F)** Immobility time in the FST. ^#^*P* < 0.05 compared with the control group; ^*^P < 0.05 compared with the CUMS group.

Male Sprague–Dawley (SD) rats with body weights of 180~200 g were purchased and housed at room temperature (25 ± 2°C, 55 ± 10% relative humidity) on a 12:12 h light–dark cycle. The rats were allowed free access to regular laboratory rat chow and water for 1 week. Following 1 week of acclimation, the rats were randomized into 4 groups (*n* = 12): control, CUMS (chronic unpredictable mild stress), PCO (dissolved in 0.01% (v/v). Tween 80 in 0.9% saline; rats received 0.8 mL/kg essential oil orally once a day), and FLX (the rats were orally administered a dosage of 10 mg/kg fluoxetine hydrochloride once a day). The control group and CUMS group were given 0.9% saline (10 mL/kg). Except for those in the control group, the rats in the other groups were exposed to CUMS for 4 weeks according to previous methods (15). The stressors included 5-min swimming in cold water (4°C), 2-min electric foot shock (rats receive unavoidable 3 mA intensity electric foot shocks for a duration of 200 ms with a frequency of one shock per second), 2-min tail clamping, 3-h noise (100 dB), subject to a hot environment (45°C) for 5 min, 24-h water deprivation, 24-h food deprivation. The above stressors were imposed separately, and each rat received no more than one stressor simultaneously. If a rat received a stressor of water deprivation or food deprivation, no more stressor was imposed to this rat at the same day. The timeline of the experiment is shown in Figure 1A.

### Open field test

Rat behavior was assessed for 5 min in an open field apparatus (122 × 122 × 45 cm), which was divided into 16 equal squares (30.5 × 30.5 cm). The rat was first placed in the center of the apparatus. The total duration (in seconds) and number of entries into the central zone were observed and counted. The apparatus was cleaned using 70% ethanol between tests (16).

### Forced swim test

Briefly, rats were put in an open cylindrical container (diameter 35 cm and height 45 cm) filled to 30-cm height with water to swim for 6 min. The latency to immobility (in seconds) was noted in the first 2 min (maximum of 120 s). The total immobility time of the rats in the last 4 min was summed to reflect the depressive-like behavior of rats. Immobility status means the absence of movement, except for motion necessary to keep the heads above the water for breathing. The swimming water was changed after each test (17).

### Sample collection and preparation

The animals were sacrificed by decapitation after the behavioral tests were completed. Feces from the colon were collected in sterile conical tubes for microbial community and SCFA analyses. The hippocampus was isolated and washed with 0.9% saline to measure the levels of 5-HT and BDNF. Blood was collected, treated with EDTA, and centrifuged, and the supernatant was used to measure the level of corticosterone. All the samples were kept at −80°C before assessment.

### Analysis of intestinal microbial community diversity

The method used for this experiment has been previously reported (18). Briefly, microbial DNA was extracted from feces. We chose the V3-V4 16S rRNA region for generating amplicons and taxonomic analysis. PCR amplicons were purified, qualified, and paired-end sequenced. The sequences were subsequently grouped into operational taxonomic units (OTUs). The differences in the gut microbial taxonomic composition were compared using Metastats software. The sequencing data of this study was uploaded to the Global Pharmacopeia Herbal Genome Database (19) and could be accessed via http://www.gpgenome.com/species/673.

### Analysis of short-chain fatty acids

This experiment was performed as described previously (20). The concentrations of SCFAs were measured by a GC-2010 gas chromatograph fitted with a DB-FFAP column (30 m × 0.25 mm × 0.25 μm; Agilent, United States). Standard solutions of acetic acid, propionic acid, butyric acid, valeric acid, and caproic acid were prepared at 1, 0.4, 0.2, 0.1, 0.05, 0.01 and 0.005 mg/ml. Each fecal sample (0.2 g) was blended in 0.8 ml of deionized water by vortexing and subsequently centrifuged at 5,000 rpm and 4°C for 20 min. A total of 450 μl of the supernatant was added to 50 μl of 50% H_2_SO_4_, vortexed and centrifuged at 12,000 rpm for 10 min, after which the mixture was incubated at 4°C for 30 min. The supernatant was collected for GC analysis.

### Measurement of the 5-HT and BDNF levels in the hippocampus

The hippocampal tissue was homogenized in 0.9% saline and centrifuged. The supernatant was collected and used for the determination of 5-HT and BDNF levels (21). The procedures were performed according to the manufacturer's instructions. Briefly, the competitive enzyme immunoassay technique was used to determine the level of 5-HT. Standards or samples were added to a microplate precoated with ST/5-HT antigen, and then, a biotin-conjugated antibody specific for ST/5-HT was added. After that, HRP-conjugated streptavidin was added to form an immune complex, and the chromogenic substrate TMB was subsequently added to produce a discernible color. The solid-phase sandwich ELISA was used to measure the amount of BDNF. Samples or standards were added to a microplate precoated with BDNF antibody. Then, HRP-conjugated streptavidin was added, and the samples were incubated and washed. Finally, the chromogenic substrate TMB was added to react with the enzyme-antibody-target complex to produce a measurable signal. The concentrations of 5-HT or BDNF in the samples were then determined by comparing the OD to the standard curve.

### Measurement of plasma corticosterone levels

The plasma corticosterone concentrations were analyzed using an immunoassay kit according to the manufacturer's protocols (22). In brief, standards or samples were added to a microplate precoated with rat corticosterone antigen. After incubation, biotin-conjugated anti-rat corticosterone antibody was added, the mixture was combined with HRP-conjugated streptavidin to form an immune complex, incubated, washed, and added to the chromogenic substrate TMB to produce a color for determination.

### Statistical analysis

Statistical analysis was performed using GraphPad Prism 8 (version 8.2.1 (441), ©1992–2019 GraphPad Software, Inc.). The data are expressed as the mean ± standard error of the mean. One-way ANOVA was applied to determine significant differences. *P* < 0.05 was considered to indicate statistical significance.

## Results

### Chemical analysis of PCO

**Figure 2:**
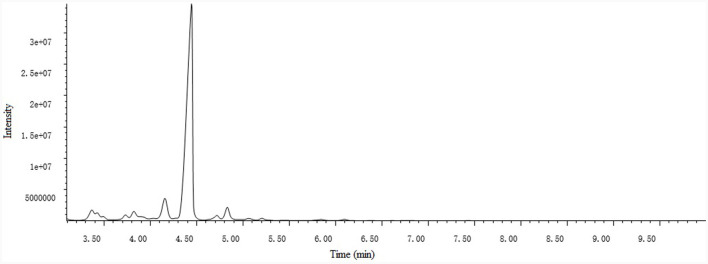
Total ions current (TIC) diagram of patchouli essential oil (PCO).

A total of 15 compounds were identified by GC-MS analysis in PCO. Figure 2 shows the total ions current (TIC) diagram. Among them, four main compounds, Ledene oxide-(II) (44.36%), 2,6,10-Dodecatrienal, 3,7,11-trimethyl-, (E, E)- (20.72%), Ethanone, 1-(2,4,6-trihydroxyphenyl)- (7.05%) and Patchouli alcohol (5.28%), together account for 77.42% of the total relative peak area (Table 1).

**Table 1:** GC-MS data of the major constituents of patchouli essential oil (PCO).

**No**.	**Retention time (min)**	**Molecular weight**	**Molecular formula**	**Identification**	**Relative percentage (%)**
1	3.424	220	C_15_H_24_O	(-)-Spathulenol	3.59
2	3.493	220	C_15_H_24_O	Caryophyllene oxide	4.35
3	3.733	220	C_15_H_24_O	Diepicedrene-1-oxide	3.55
4	3.825	222	C_15_H_26_O	Globulol	3.06
5	4.157	222	C_15_H_26_O	Epiglobulol	1.77
6	4.414	222	C_15_H_26_O	Patchouli alcohol	5.28
7	4.546	220	C_15_H_24_O	Ledene oxide-(II)	44.36
8	4.723	222	C_15_H_26_O	2,6,10-Dodecatrien-1-ol, 3,7,11-trimethyl-	2.26
9	4.838	168	C_8_H_8_O_4_	Ethanone, 1-(2,4,6-trihydroxyphenyl)-	7.05
10	5.061	220	C_15_H_24_O	2,6,10-Dodecatrienal, 3,7,11-trimethyl-, (E, E)-	20.72
11	5.198	236	C_16_H_28_O	(-)-Isolongifolol, methyl ether	2.44
12	5.296	204	C_15_H_24_	α-Guaiene	0.44
13	5.845	220	C_15_H_24_O	Aromadendrene oxide-(2)	0.31
14	6.022	230	C_16_H_22_O	1,4-Hexadien-3-one, 5-methyl-1-[2,6,6-trimethyl-2,4-cyclohexadien-1-yl]-	0.53
15	6.102	254	C_15_H_26_O_3_	Perhydrocyclopropa[e]azulene-4,5,6-triol, 1,1,4,6-tetramethyl	0.27

### PCO alleviated anxiety- and depressive-like behaviors caused by CUMS

In the OFT, the administration of PCO reversed both the decreased number of entries and time spent in the center zone in CUMS rats. These indicators are associated with anxiety-related behaviors (Figures 1B, C). Figure 1D shows representative track sheets from the OFT. The FST data revealed that after 4 weeks of CUMS, there was a significant decrease in the latency to immobility and an increase in the immobility time compared to those in the control group. These findings suggest that the rats exhibited a depressive-like state. PCO treatment significantly reversed these two changes, indicating its antidepressant-like effect (Figures 1E, F).

### PCO increased the abundance of beneficial microbes and reversed intestinal flora disorder caused by CUMS

**Figure 3:**
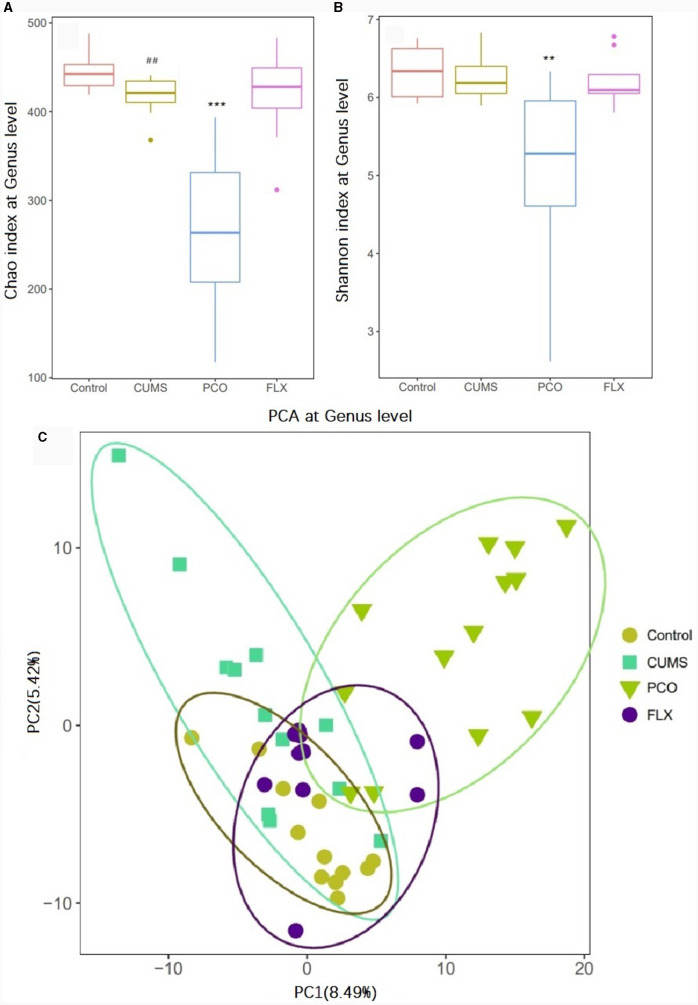
Effects of patchouli essential oil (PCO) on the α diversity **(A, B)** and β diversity **(C)** of the gut microbiota at the genus level. ^*##*^*P* < 0.01 compared with the control group; ^**^P < 0.01 and ^***^P < 0.001 compared with the chronic unpredictable mild stress (CUMS) group.

16S rRNA sequencing analysis yielded a total of 4,539,206 raw reads. We identified 9 phyla, 14 classes, 18 orders, 35 families, 119 genera, and 118 species in the four groups that were considered qualified. The PCO group exhibited significant decreases in the Chao1 index and Shannon index of alpha diversity (*P* < 0.001*, P* < 0.01) compared to those in the CUMS group (Figures 3A, B). Additionally, beta diversity-based principal component analysis (PCA) demonstrated that the microbial community composition in the PCO group differed from that in the CUMS group, as indicated by the clustering of the samples in the plots (Figure 3C).

**Figure 4:**
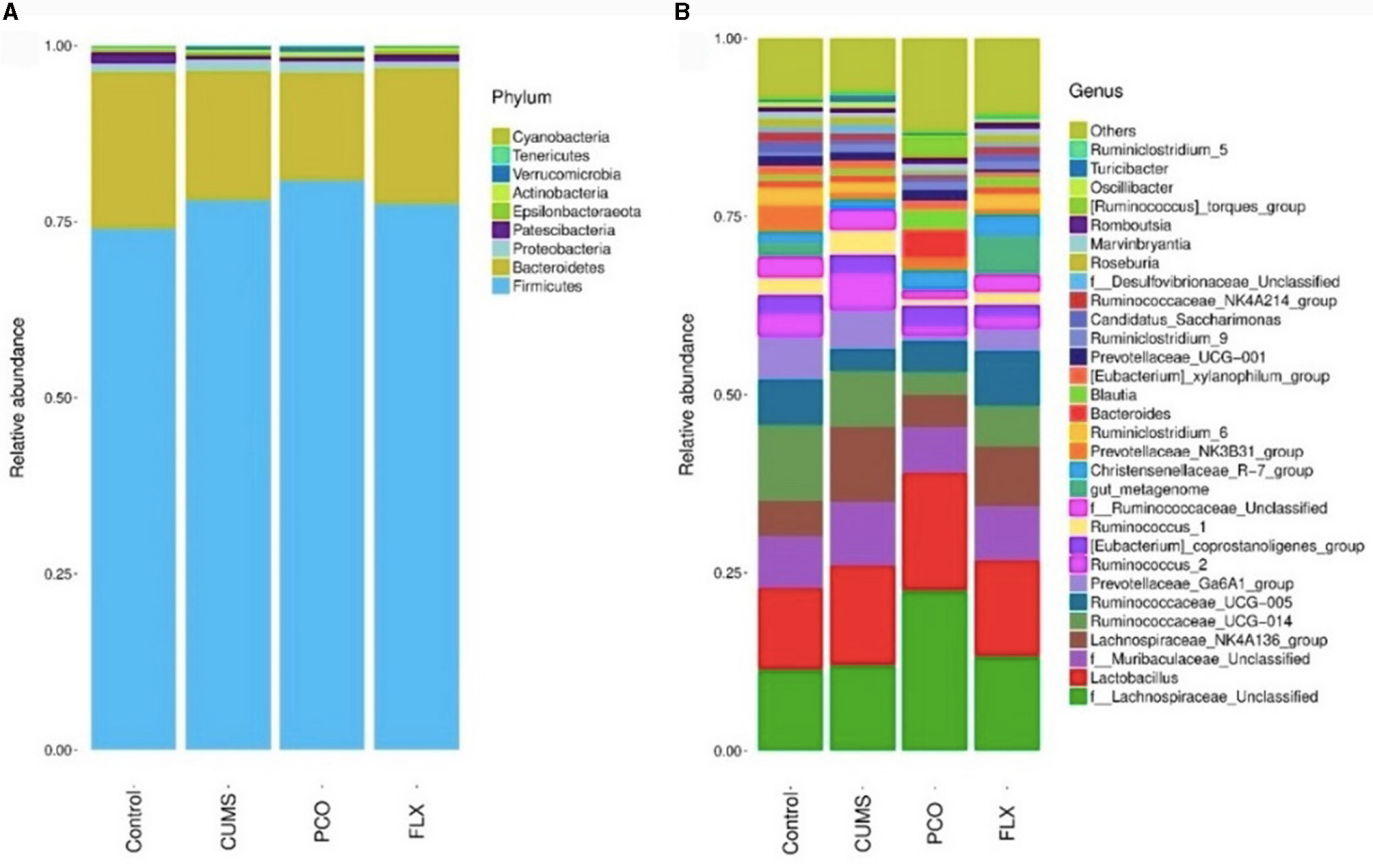
Changes in bacterial taxa caused by patchouli essential oil (PCO). **(A)** Phylum level. **(B)** Genus level.

Metastats analysis revealed that at the phylum level, the dominant microbial taxa were Firmicutes and Bacteroidetes (Figure 4A). Additionally, *Lactobacillus* was identified as the most abundant microbial taxon at the genus level (Figure 4B).

**Figure 5:**
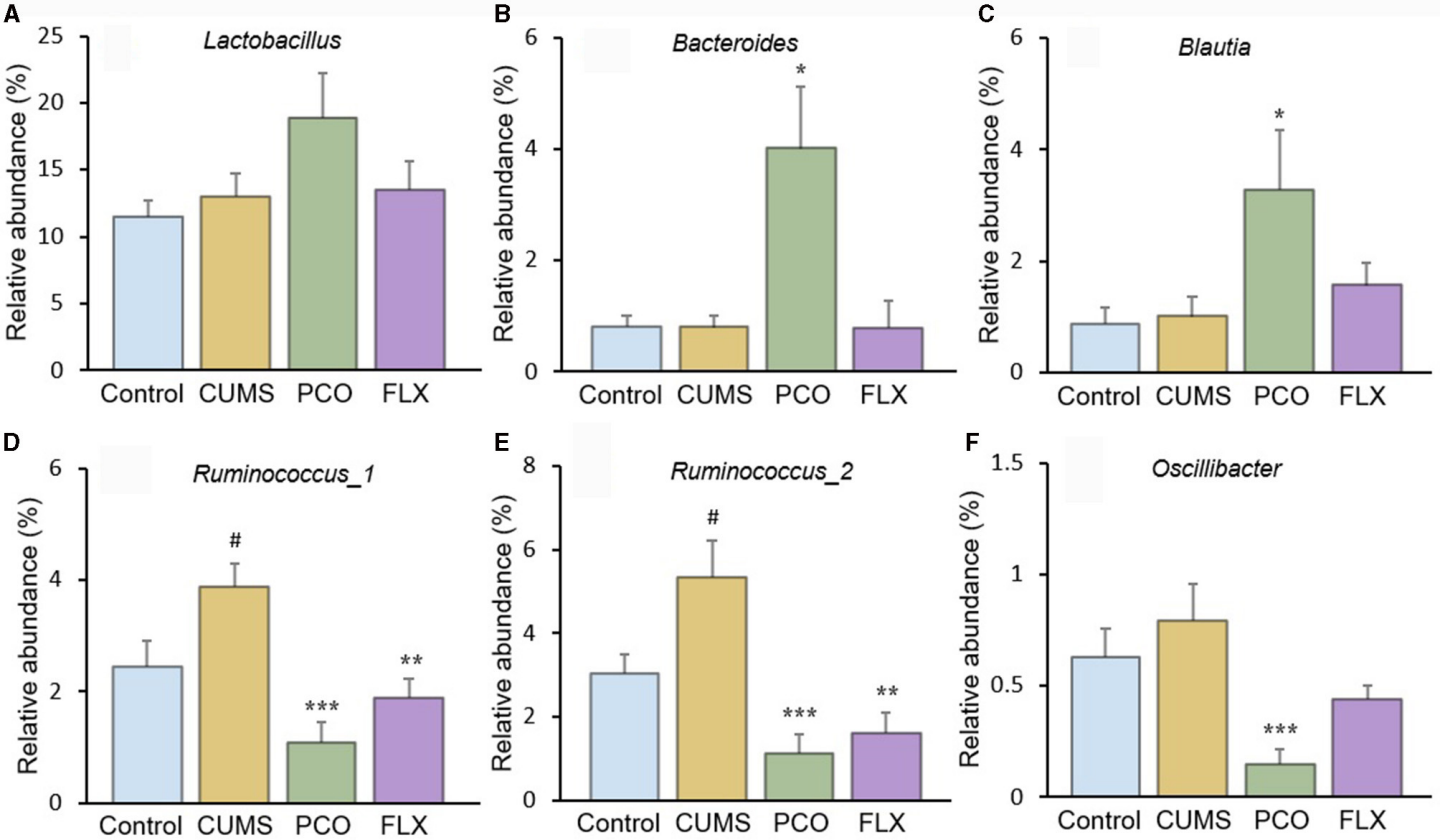
Relative abundances of specific genera affected by patchouli essential oil (PCO). **(A)** *Lactobacillus*, **(B)** *Bacteroides*, **(C)** *Blautia*, **(D)** *Ruminococcus_1*, **(E)** *Ruminococcus_2*, and **(F)** *Oscillibacter*. ^#^*P* < 0.05 compared with the control group; ^*^P < 0.05, ^**^P < 0.01 and ^***^P < 0.001 compared with the chronic unpredictable mild stress (CUMS) group.

Although there were no significant differences between the control group and the CUMS group, the PCO group exhibited greater relative abundances of *Bacteroides* (Figure 5B) and *Blautia* (Figure 5C) than did the CUMS group. The relative abundance of *Lactobacillus* (Figure 5A), which was the most abundant genus identified, was not significantly increased by PCO (*P*>0.05). CUMS changed the gut microbial communities to have significantly greater relative abundances of *Ruminococcus_1* (Figure 5D) and *Ruminococcus_2* (Figure 5E). The PCO group presented lower relative abundances of *Ruminococcus_1, Ruminococcus_2*, and *Oscillibacter* (Figure 5F) than the CUMS group.

### PCO recovered SCFA disorder caused by CUMS

**Figure 6:**
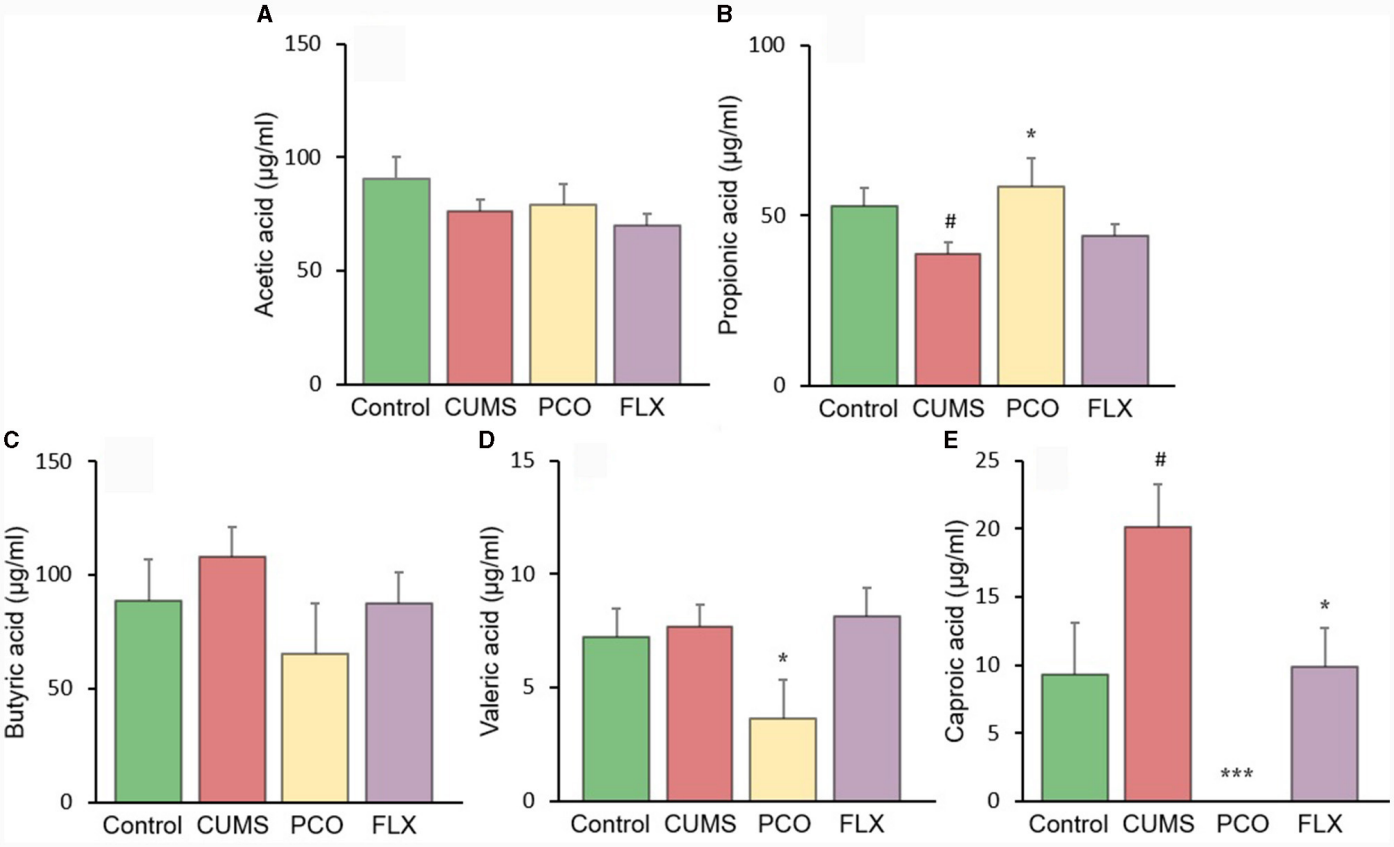
Changes in short-chain fatty acid (SCFA) levels in fecal samples. **(A)** acetic acid, **(B)** propionic acid, **(C)** butyric acid, **(D)** valeric acid, and **(E)** caproic acid. ^#^*P* < 0.05 compared with the control group; ^*^P < 0.05 and ^***^P < 0.001 compared with the chronic unpredictable mild stress (CUMS) group.

The changes in SCFA levels in the rat fecal samples are illustrated in Figure 6. The levels of acetic acid and butyric acid remained unchanged by CUMS or PCO. However, compared with that in the control group, the concentration of propionic acid in the CUMS group decreased, while the concentration of caproic acid increased. In contrast, compared with the CUMS group, PCO significantly increased the level of propionic acid and decreased the level of caproic acid to the point where it was undetectable. CUMS had no effect on valeric acid, but PCO significantly decreased its level compared to that in the CUMS group.

### PCO restored 5-HT dysfunction caused by CUMS but not the changes in BDNF or corticosterone

**Figure 7:**
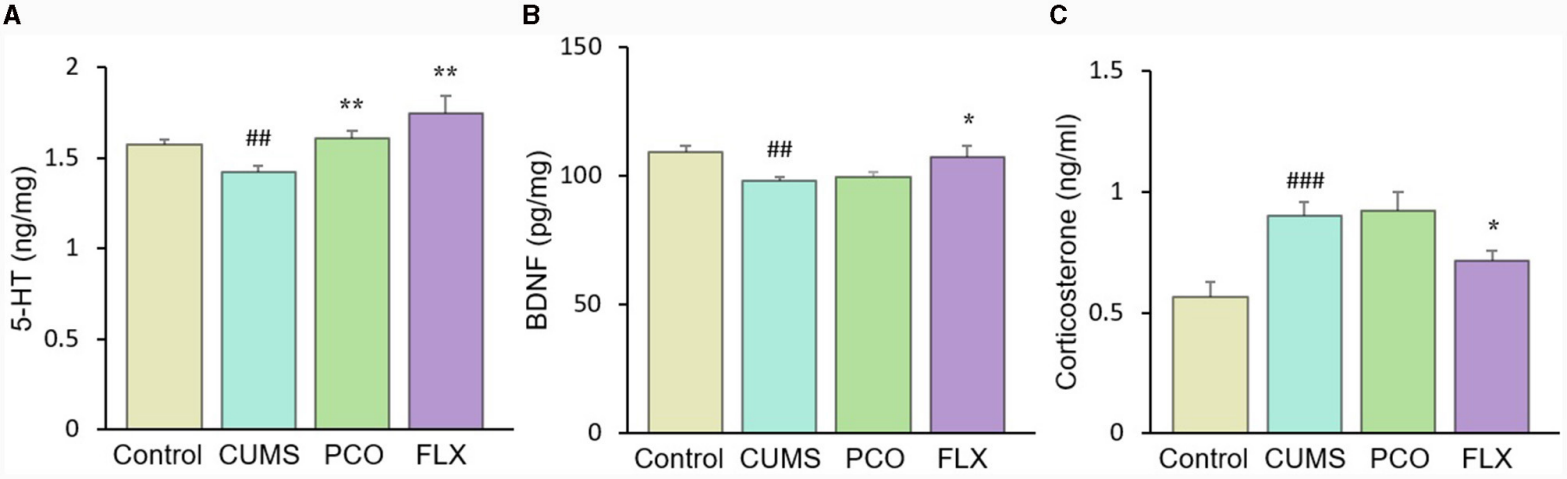
Changes in **(A)** 5-HT and **(B)** brain-derived neurotrophic factor (BDNF) levels in the hippocampus and **(C)** corticosterone levels in plasma. ^*##*^*P* < 0.01 and ^*###*^*P* < 0.001 compared with the control group; ^*^P < 0.05 and ^**^P < 0.01 compared with the chronic unpredictable mild stress (CUMS) group.

**Figure 8:**
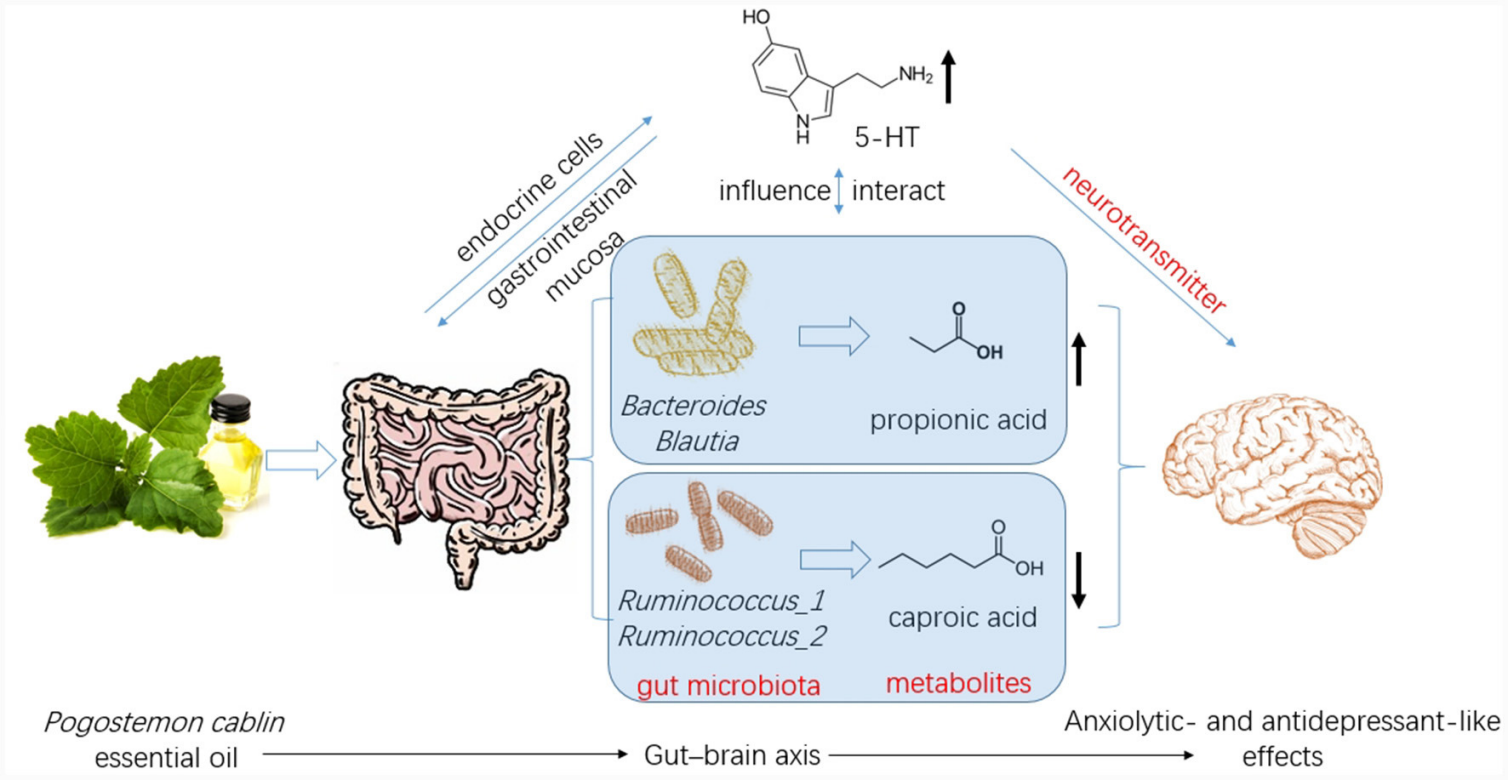
Patchouli essential oil (PCO) improves stress-related anxiety- and depression-like behaviors through gut-brain axis.

CUMS resulted in a significant reduction in 5-HT levels in the hippocampus, which was significantly restored by PCO or FLX treatment (Figure 7A). Similarly, BDNF levels were significantly lower in the CUMS group than in the control group; however, PCO did not significantly affect BDNF levels, whereas FLX increased BDNF concentrations in the hippocampus (Figure 7B). Additionally, CUMS significantly increased the basal corticosterone concentration, which was attenuated by FLX treatment (Figure 7C). PCO did not influence the corticosterone concentration. A schema diagram demonstrating the hypothetical mechanisms of anxiolytic- and antidepressant-like effects of PCO is shown in Figure 8.

## Discussion

Depression is a chronic and debilitating disorder that is receiving increasing attention for its association with long-term stress (23). The CUMS model, a classical model of depression, is considered to be more similar to the pathogenesis of human depression. Anxiety, on the other hand, is a normal response to threats or psychological stress (24). CUMS can also be used to study anxiolytic activity, as it induces the development of anxious behaviors in animals (25–29). We provide new evidence that PCO (the intervention studied here) can prevent the development of anxiety- and depressive-like behaviors induced by CUMS (the stressor). Furthermore, our findings suggest that the effects of PCO may be attributed to its ability to modulate the gut microbiota.

In this study, we identified several changes that occurred in the gut microenvironment. While CUMS did not have any significant effects, we observed that the consumption of PCO had a positive impact on the relative abundances of *Bacteroides* and *Blautia*, which are known as probiotics. Additionally, we found that the relative abundance of *Lactobacillus* was greater after the administration of PCO (*P*>0.05). The results of this study are consistent with previous findings suggesting that PCO has a prebiotic-like effect (30). Prebiotics play a significant role in the development and management of anxiety and depression (31). Recent reports have shown that oral treatment with *Bacteroides* can reverse anxiety-like behaviors in mice, as well as correct gut permeability and abnormal intestinal cytokine profiles (32). Strains of *Blautia* and *Lactobacillus* have been found to attenuate anxiety- and depressive-like behaviors (33, 34). Short-chain fatty acids (SCFAs) play crucial roles in both digestion and central functions and also have significant effects on various behaviors (35). Species of *Bacteroides* and *Blautia* are primary producers of acetic acid and propionic acid in the human gut. Acetic acid is believed to have the ability to prevent gut infections and maintain the integrity of the gut barrier (36). Propionic acid has also been shown to play a role in maintaining proper intestinal permeability (37). Propionic acid levels are significantly decreased in depressed animals (38). Here, we found a similar result, which was reversed by PCO. Our results demonstrated that PCO might exert anxiolytic- and antidepressant-like effects by modulating probiotic bacteria, including *Bacteroides, Blautia*, and even *Lactobacillus* species.

*Ruminococcus* is common in the human gut microbiota. Antidepressants reduce the abundance of *Ruminococcus*, which is causally related to their antidepressant effects (39). Our results indicated that both PCO and FLX decreased the levels of *Ruminococcus-1* and *Ruminococcus-2*, which were significantly increased by CUMS. Therefore, the present study suggested that *Ruminococcus-1* and *Ruminococcus-2* may play roles in depressive behaviors, which aligns with the findings of previous research indicating a change in the relative abundance of *Ruminococcus*. The *Ruminococcus bacterium* strain is highly prolific in its ability to produce caproic acid (40). Caproic acid also partly contributes to depressive phenotypes through the gut–brain axis (41). Valeric acid may play a role in the pathological process of depression due to its resemblance to GABA (42). Caproic acid levels were significantly increased in the CUMS group but not detectable in the PCO group. Additionally, the valeric acid concentration was lower in the PCO group than in the CUMS group. Taken together, these findings suggest that *Ruminococcus* and its metabolite SCFAs, especially caproic acid, may be important segments of PCO-related gut microbiota-brain interactions. The genus *Oscillibacter* is also significantly associated with depression (42). The main metabolic end product of the *Oscillibacter* type strain is valeric acid (42). Our results suggest that *Oscillibacter*, like *Ruminococcus*, may be the bacteria responsible for mediating the antidepressant-like effect of PCO.

Brain 5-HT deficiency is considered to be a significant pathogenic mechanism in depression (41). The neurotransmitter 5-HT also plays significant roles in stress and anxiety (36). The restored level of 5-HT in the hippocampus of rats exposed to CUMS and treated with PCO may explain the anxiolytic- and antidepressant-like effects of PCO. The majority of human 5-HT is synthesized by specific gut endocrine cells. Additionally, the presence of certain gut bacteria can influence the biosynthesis of 5-HT (43). Furthermore, 5-HT can influence the maintenance of the gastrointestinal mucosa (36). Gut-derived fatty acids can cross the blood–brain barrier (BBB), interact with synaptic neurotransmitters, and influence behaviors (32). The results here confirmed that the central nervous system effects of PCO are associated with interactions with the gut microbiota.

## Conclusion

In the present study, the impact of PCO on stress-sensitive behaviors was investigated using a rat model of CUMS. Our results suggest that PCO may have anxiolytic and antidepressant effects. These effects may be mediated by modulation of the gut microbiota and SCFAs, which in turn affect 5-HT levels in the brain via the gut–brain axis. These findings have important implications for the potential therapeutic use of PCO in the fields of medicine and health.

## Data availability statement

The raw data supporting the conclusions of this article will be made available by the authors, without undue reservation.

## Ethics statement

The animal study was approved by the Ethics Committee of Guangdong Provincial Hospital of Chinese Medicine. The study was conducted in accordance with the local legislation and institutional requirements.

## Author contributions

PO: Funding acquisition, Methodology, Resources, Writing—original draft. DK: Conceptualization, Funding acquisition, Supervision, Writing—original draft, Writing—review & editing. WY: Data curation, Formal analysis, Funding acquisition, Methodology, Validation, Writing—review & editing. XS: Methodology, Project administration, Writing—review & editing. XM: Investigation, Methodology, Project administration, Supervision, Writing—review & editing. YL: Formal analysis, Methodology, Writing—review & editing.
